# Anti-Cancer Potential of MAPK Pathway Inhibition in Paragangliomas–Effect of Different Statins on Mouse Pheochromocytoma Cells

**DOI:** 10.1371/journal.pone.0097712

**Published:** 2014-05-20

**Authors:** Stephanie M. J. Fliedner, Tobias Engel, Nikoletta K. Lendvai, Uma Shankavaram, Svenja Nölting, Robert Wesley, Abdel G. Elkahloun, Hendrik Ungefroren, Angela Oldoerp, Gary Lampert, Hendrik Lehnert, Henri Timmers, Karel Pacak

**Affiliations:** 1 Section on Medical Neuroendocrinology, Program in Reproductive and Adult Endocrinology, Eunice Kennedy Shriver National Institute of Child Health and Human Development, National Institutes of Health, Bethesda, Maryland, United States of America; 2 1st Department of Medicine, University Medical Center Schleswig-Holstein, Lübeck, Germany; 3 Department of Endocrinology, Radboud University Nijmegen Medical Centre, Nijmegen, The Netherlands; 4 Radiation Oncology Branch, National Cancer Institute, National Institutes of Health, Bethesda, Maryland, United States of America; 5 Department of Endocrinology, William Harvey Research Institute and Barts Cancer Institute, Barts and the London School of Medicine, Queen Mary University of London, London, United Kingdom; 6 Warren G. Magnuson Clinical Center, National Institutes of Health, Bethesda, Maryland, United States of America; 7 Cancer Genetics Branch, National Human Genome Research Institute, National Institutes of Health, Bethesda, Maryland, United States of America; 8 Pompano Beach, Florida, United States of America; Dana-Farber Cancer Institute, United States of America

## Abstract

To date, malignant pheochromocytomas and paragangliomas (PHEOs/PGLs) cannot be effectively cured and thus novel treatment strategies are urgently needed. Lovastatin has been shown to effectively induce apoptosis in mouse PHEO cells (MPC) and the more aggressive mouse tumor tissue-derived cells (MTT), which was accompanied by decreased phosphorylation of mitogen-activated kinase (MAPK) pathway players. The MAPK pathway plays a role in numerous aggressive tumors and has been associated with a subgroup of PHEOs/PGLs, including *K-RAS-*, *RET-*, and *NF1*-mutated tumors. Our aim was to establish whether MAPK signaling may also play a role in aggressive, succinate dehydrogenase (SDH) B mutation-derived PHEOs/PGLs. Expression profiling and western blot analysis indicated that specific aspects of MAPK-signaling are active in SDHB PHEOs/PGLs, suggesting that inhibition by statin treatment could be beneficial. Moreover, we aimed to assess whether the anti-proliferative effect of lovastatin on MPC and MTT differed from that exerted by fluvastatin, simvastatin, atorvastatin, pravastatin, or rosuvastatin. Simvastatin and fluvastatin decreased cell proliferation most effectively and the more aggressive MTT cells appeared more sensitive in this respect. Inhibition of MAPK1 and 3 phosphorylation following treatment with fluvastatin, simvastatin, and lovastatin was confirmed by western blot. Increased levels of CASP-3 and PARP cleavage confirmed induction of apoptosis following the treatment. At a concentration low enough not to affect cell proliferation, spontaneous migration of MPC and MTT was significantly inhibited within 24 hours of treatment. In conclusion, lipophilic statins may present a promising therapeutic option for treatment of aggressive human paragangliomas by inducing apoptosis and inhibiting tumor spread.

## Introduction

Recently, lovastatin has been suggested as promising potential therapeutic option to treat RET, NF1, and TMEM127 mutation-derived catecholamine producing adrenal and extra-adrenal chromaffin cell tumors (pheochromocytomas (PHEOs) and paragangliomas (PGLs), respectively) [1]. However, the risk for metastatic disease in these tumors is relatively low and tumor resection is thus almost always curative. In contrast, the risk for malignant PHEOs/PGLs is particularly high in the case of succinate dehydrogenase B (SDHB) gene mutations [2], [3], and novel treatment strategies are urgently needed for this condition.

A recent New England Journal of Medicine Article reported decreased cancer-related mortality in patients who were prescribed statins previous to diagnosis [4]. In agreement with this, numerous *in vitro* and *in vivo* studies demonstrated anti-cancer effects of lovastatin and other statins alone or as part of a combined treatment regimen for aggressive tumors (reviewed in [5]–[7]). At higher concentrations than are required for reduction of cholesterol levels, statins have been shown to inhibit the mevalonate pathway severely enough to inhibit the synthesis of isoprenoids, which act as necessary membrane anchors for proper function of certain proteins [8]. The anti-cancer effects of statins have mainly been associated with inhibition of Ras-prenylation (i.e. farnesylation or geranylgeranylation), which among other effects, disrupts activation of downstream players in the MAPK pathway [9], [10]. Over-activation of the mevalonate pathway, including MAPK-signaling has been shown to be sufficient for cell transformation [11]. Excessive MAPK signaling supports apoptosis inhibition, proliferation, and migration and is a key characteristic of many cancer types (summarized in [12], [13]). In case of PHEOs/PGLs, elevated MAPK pathway activity is apparent in *H-RAS*, *K-RAS*, *TMEM127*, *RET*, *NF1*, and possibly *MAX* mutation-related tumors [14]–[22].

To our knowledge, currently no evidence for increased MAPK signaling in SDHB-derived PGLs has been presented. In cells from patients with Cowden-like syndrome and SDHB or D gene variants, however, increased levels of MAPK 1 and 3 phosphorylation have been observed [23]. In the presence of an active MAPK pathway, statins may provide a promising treatment or co-treatment option for currently fatal malignant PGLs.

The extent of the anti-cancer effects has been shown to vary depending on model and type of statin used [24]–[30]. Thus, we evaluated which of the seven currently available statins may be most effective for PHEO/PGL treatment.

## Materials and Methods

Fluvastatin, pravastatin, lovastatin, and simvastatin were all obtained from Cayman Chemicals (Ann Arbor, MI); atorvastatin and rosuvastatin were obtained from Enzo Life Sciences, Inc. (Farmingdale, NY).

### Cell culture

Mouse tumor tissue-derived (MTT) cells have been recently developed in our lab [31]. All animal studies necessary for development and characterization of MTT cells were conducted in accordance with the principles and procedures outlined in the National Institute of Health Guide for the Care and Use of Animals, and approved by the *Eunice Kennedy Shriver* National Institute of Child Health and Human Development Animal Care and Use Committee (Protocol number ASP# 06-028). MTT cells are property of the NIH. Mouse pheochromocytoma cells (MPC 4/30PRR) were a generous gift from Dr. Tischler, TUFTS, Boston. Cells were maintained in DMEM (Gibco, Life Technologies, Grand Island, NY), supplemented with 10% heat-inactivated horse serum (Hyclone Logan, UT), 5% fetal bovine serum (Gibco), HEPES (Gibco), and penicillin (10,000 units/ml)/streptomycin (100,000 µg/ml) (Gibco) in a humidified atmosphere containing 5% CO_2_ at 37°C. Medium was changed every other day and cells were passaged when 80-90% confluence was reached. All statins were compared to the appropriate concentration of vehicle (DMSO). When statins were combined to evaluate potential additive effects, 25 µM of 2 different statins were compared to 50 µM of each of the individual statins.

### Proliferation assays

Cells were seeded into collagen coated 96-well plates (BD Biosciences, San Jose, CA) at 10,000 cells per well, and allowed to attach for 24 h. Then media was exchanged with concentrations of 6.25, 12.5, 25, and 50 µM of the different statins, dissolved in supplemented media. After 0, 24, 48, and 72 h of treatment, cell proliferation was assessed with the Cell Proliferation Kit II (XTT) (Roche applied science; Indianapolis, IN) according to the product manual. After four hours of incubation, the plates were measured at 490 nm with 650 nm reference wavelength in a microplate reader (Victor^3^ 1420 multilabel counter, Perkin Elmer, Waltham, MA). All experiments were performed in quadruplicate and repeated at least twice.

### Spontaneous cell migration assays

Cells were seeded at 60,000 cells per well for spontaneous migration assessment in an xCELLigence DP device (Roche Diagnostics, Mannheim, Germany) as previously reported [32]. The spontaneous migration was recorded for 24 hours in the presence of 5 µM fluvastatin, lovastatin, and simvastatin alone, or in combination with 100 µM trans, trans farnesol (Sigma-Aldrich Co., St. Louis, MO, USA).

### MAPK pathway gene-enrichment in SDHB PGLs

Expression data was extracted from previously presented microarray data on 45 samples of pseudohypoxic PHEOs/PGLs, including 18 SDHB, 8 SDHD head and neck (HN), 6 SDHD abdominal and thoracic (AT), and 13 VHL samples compared to normal adrenal medulla [33]. As reported, prediction analysis of microarray identified 6937 genes as characteristic for one of the different subgroups of pseudohypoxic PHEOs/PGLs. These were mapped against a list of 254 MAPK pathway genes (Kyoto Encyclopedia of Genes and Genome Pathway). Predominant over-expression in SDHB samples compared to normal medulla was confirmed by ANOVA and intervals were based on the Studentized range statistic, Tukey's “Honest Significant Difference” method.

### Ethics Statement

Tissue collection was approved by the institutional review board of the *Eunice Kennedy Shriver* National Institutes of Child Health and Human Development. Written informed consent was obtained from each patient.

### Human tumor tissue

PHEO/PGL tissue was immediately frozen upon resection. Patient and tumor information for each sample are presented in Table 1. Frozen samples were homogenized on ice in Tissue Protein Extraction Reagent (Thermo Fisher Scientific Inc., Lafayette, CO, USA) with 0.1% phosphatase inhibitor (Cell Signaling Technology Inc., Danvers, MA, USA) and one protease inhibitor Complete Mini tablet per 10 ml solution (Roche Applied Science, Indianapolis, IN, USA). Homogenates were centrifuged at 10,000×g for 5 minutes at 4°C. The supernatant was used as protein extract.

**Table 1:** Patient information.

Sample ID	Gender	Age at resection	Location	Mutation	Characteristic
B1	M	38.2	E	SDHB	PM
B2	F	11.4	E	SDHB	P
B3	M	53.1	E	SDHB	P
B4	M	44.2	E	SDHB	MM
B5	M	34.1	E	SDHB	MM
B6	F	42.3	E	SDHB	MM
D1	F	44.6	HN	SDHD	P
D2	M	39.6	HN	SDHD	P
D3	F	11.8	HN	SDHD	P
N1	M	17.0	A	NF1	P
N2	M	26.6	A	NF1	P
M1	unknown	unknown	A	-	N
M2	unknown	unknown	A	-	N

Abbreviations: A: adrenal, E: extra-adrenal, F: female, HN: head and neck, ID: identifier, M: male, MM: metastatic metastases, N: normal, P: primary non-metastatic, PM: primary metastatic.

### Cell harvesting

Cells were seeded in collagen coated plates [34] at 600,000 cells/ml and allowed to attach for a minimum of 18 h before media was changed to 25 µM statin solutions or DMSO control. Treatment solutions were renewed after 24 h. After 48 h of treatment, cells were collected with a rubber policeman and washed 3 times in ice cold PBS by centrifugation at 500×g. Cells were collected at 1500×g, dissolved in 0.1% cholamidopropyldimethylammoniopropanesulfate, containing one protease inhibitor Complete Mini tablet per 7 ml solution (Roche Applied Science) and 0.5% Phosphatase Inhibitor Cocktail 2 (Sigma-Aldrich Co.). Samples were sonicated for 2 min on ice and subsequently centrifuged at 10,000×g for 10 min, 4°C. The supernatant was used as protein extract.

### Western blot

To estimate protein concentrations the Quant-iT Protein Assay Kit (Invitrogen, Life Technologies Corporation) was used. Equal protein amounts of tumor extracts in PAGE gel LDS sample buffer (Thermo Fisher Scientific) or cell extracts in Lämmli buffer were loaded onto 4–20% Criterion TGX precast gels (Bio-Rad Laboratories, Hercules, CA, USA) or hand cast 12% gels, separated by sodium-dodecylsulfate polyacrylamide gel electrophoresis, and transferred to PVDF membranes (Immobilon-P, EMD Millipore Corporation, Billerica, MA, USA). Membranes were blocked in 5% non-fat dry milk in phosphate buffered saline (pH 7.4) with 0.01–0.05% Tween 20 (Sigma-Aldrich Co.) for 1 hour. Antibodies were dissolved in 5% bovine serum albumin or 5% non-fat dry milk in tris-buffered saline (pH 7.6) with 0.01–0.05% Tween 20. Membranes were incubated with primary antibodies overnight at 4 °C or for 1 h at room temperature. Primary antibodies were rabbit anti-cleaved-caspase-3, rabbit anti-p44/p42 MAPK, rabbit anti-phospho-p44/p42 MAPK, rabbit anti-PARP, rabbit anti-GAPDH (Cell Signaling, Technology Inc.), mouse anti-β-actin (Sigma-Aldrich). Secondary antibodies were HRP-linked donkey anti-rabbit (GE Healthcare, Pittsburgh, PA, USA), goat anti-rabbit, and goat anti-mouse (both DAKO North America, Inc., Carpinteria, CA). Membranes were incubated in Super Signal West Pico Chemiluminescent Substrate (Thermo Fisher Scientific) and exposed to High Performance Chemiluminescence film (GE Healthcare) or incubated in Amersham ECL Prime Western Blotting Detection Reagent (GE Healthcare) and visualized in a ChemiDoc system (Bio-Rad Laboratories). Membranes were stripped with Restore PLUS Western Blot Stripping Buffer (Thermo Fisher Scientific) before re-blocking and incubation with another secondary antibody.

### Statistical analysis

A two-step approach was used to assess the anti-proliferative efficacy of each drug to decrease cell viability. In the first step, each of the 6 drugs was compared separately to vehicle for each level of treatment duration, dose, and cell type. The data used for these analyses were the (replicated) ratios of the drug well values divided by the mean of the associated set of DMSO control values. These ratios, from 2 to 4 independent experiments, were analyzed using a two-way fixed-effects ANOVA. The resulting p-values were Bonferroni-corrected by multiplying them by 24, the number of configurations of duration, dose, and cell type for a given drug. Next, drugs that overall significantly decreased proliferation were selected and the most effective durations and doses for each drug for each cell type were assessed: by an initial ANOVA, followed by ranking on the basis of post-ANOVA means, followed by Student-Newman-Keuls (SNK) post hoc tests to determine which means differed significantly. The overall performances of the 3 best performing drugs were compared using a 4-way ANOVA (factors: drug, duration, dose, and cell type); again SNK post hoc tests were done to determine which drugs were overall significantly different.

The effect of combined statins compared to each individual statin was evaluated in an analogous manner. Data are presented as the mean and standard error (based on the ANOVA and the delta-method) of at least two independent experiments. Statistical calculations were performed in Stata (Release 12, StataCorp., College Station, Texas, USA).

## Results

### Evidence for MAPK-signaling in SDHB PHEOs/PGLs

**Figure 1:**
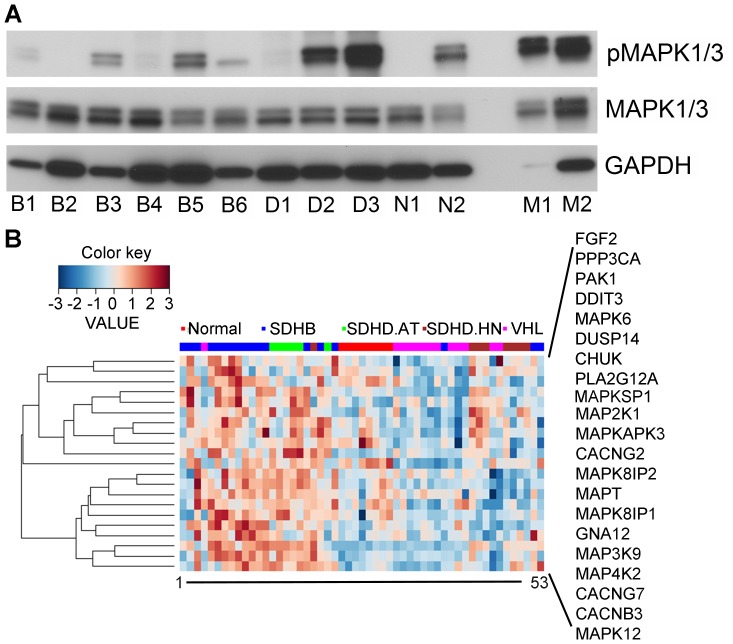
MAPK pathway representation in SDHB-derived PHEOs/PGLs. A. Western blot of pMAPK1/3, total MAPK1/3, and GAPDH in human PHEOs/PGLs. Patient and tumor information for each sample are presented in Table 1. Abbreviations: B) SDHB, D) SDHD, N) NF1, M) normal adrenal medulla. B. Heatmap showing expression of 21 MAPK pathway genes in pseudohypoxic PHEOs/PGLs. Expression of these 21 genes was significantly elevated in SDHB compared to normal medulla (p<0.002). Each sample was assigned a number. The corresponding sample identifier from the original article [33] is given in Table S1. Patient information and link to deposited data are given in the original article.

As previously demonstrated, lovastatin exerted a strong anti-proliferative effect on MPC and MTT cells, which was associated with decreased phospho-MAPK 1 and 3 (pMAPK1/3) levels [1]. To evaluate whether statin treatment may be of benefit to patients with metastatic PGLs, we assessed whether pMAPK1/3 was present in the aggressive SDHB-derived PGLs and other hereditary PHEOs/PGLs (Fig. 1A). Patient and tumor information for each sample are presented in Table 1. Among the human PHEOs/PGLs the pMAPK1/3 levels were highly variable. Although the average pMAPK1/3 levels were comparably low in SDHB tumors relative to other tested samples, phosphorylation was evident in 4 out of six SDHB samples. No difference between primary SDHB PGLs and SDHB-metastases was evident.

In addition, we evaluated the mRNA expression profile of MAPK pathway genes in pseudohypoxic PHEOs/PGLs. Mapping of 254 MAPK pathway genes to 6937 previously identified genes of interest [33] revealed a match of 85 genes. Hierarchical clustering of those 85 genes revealed two gene clusters, one of which contained 21 genes that appeared to be more highly expressed in SDHB and SDHD-AT PHEOs/PGLs compared to normal adrenal medulla and SDHD-HN and VHL PHEOs/PGLs (Fig. 1B). A closer look at these 21 genes revealed overall increased expression in the SDHB samples compared to normal adrenal medulla (p<0.002) (Fig. 1B) and an ANOVA of the individual genes with post-hoc evaluation confirmed significantly higher expression of MAPK12, CACNB3, CACNG7, MAP4K2, MAP3K9, and MAPK6 in SDHB PHEOs/PGLs than normal adrenal medulla (p≤0.02). Thus, certain aspects of MAPK signaling appear to be activated in the most aggressive SDHB-derived PHEOs/PGLs, so targeting this pathway may be a promising new treatment approach.

### Comparison of *in vitro* efficacies of different statins

**Figure 2:**
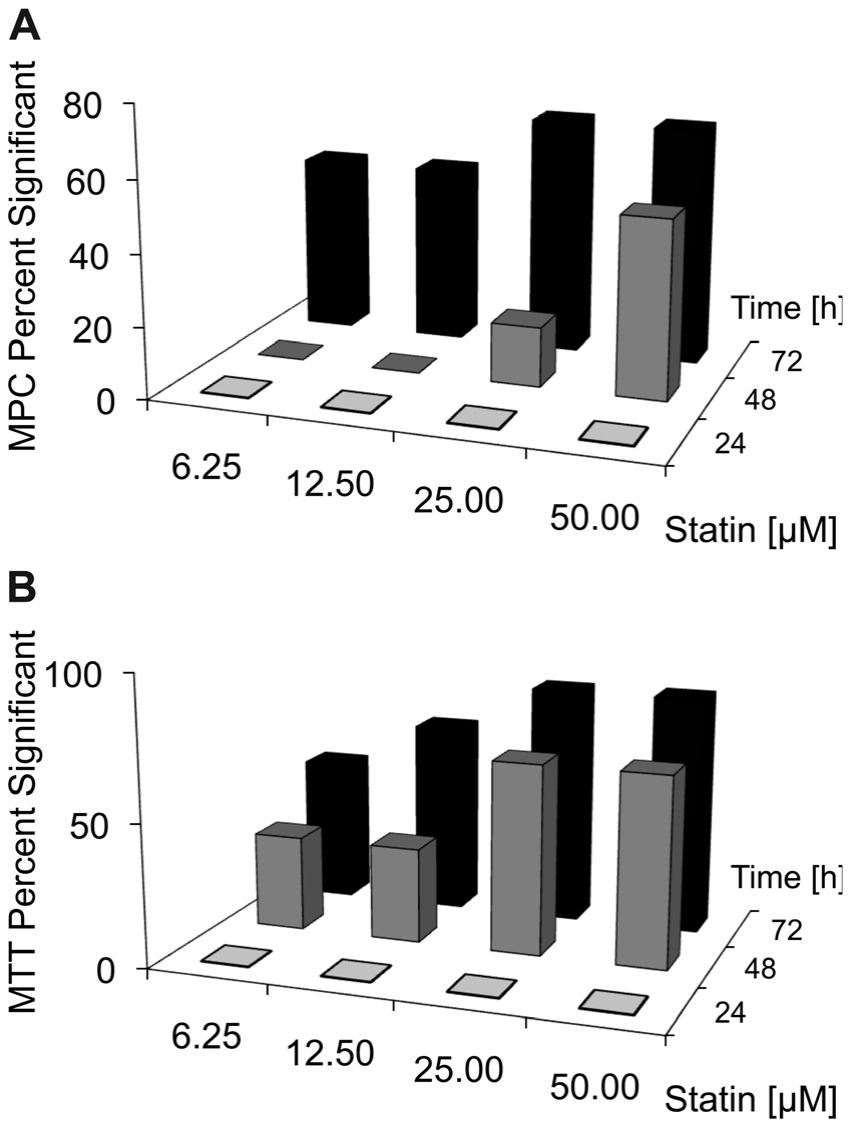
Effect of 6.25–50 µM statin treatment for 3 days on MPC and MTT proliferation. Percent significant results over all statins for all treatment doses at the different treatment durations for MPC (A) and MTT (B). The percentage of significant results was increased in MTT compared to MPC after 48 and 72 h, suggesting that MTT are more sensitive to statin treatment.

To determine the efficiency of different statins on PHEOs/PGLs, we used two mouse models; MTT and MPC cell lines. No evidence for succinate dehydrogenase dysfunction or subunit mutation is expected in MPC or MTT cells, however currently no better in-vitro model exists to study the effect of new therapeutic options for PHEOs/PGLs. Relative cell viability decreased with increasing treatment duration and concentration of the different statins for MPC and MTT. Duration of three days of treatment with the highest concentration of 50 µM was most effective for all statins (Fig. 2) and showed a significantly higher effect compared to the same dose and statin on the second day of treatment (SNK p≤0.029 for all statins except pravastatin, which was ineffective).

The occurrence of significantly decreased relative viability compared to vehicle increased with dose and treatment durations and these effects were more pronounced for MTT than MPC, indicating a higher susceptibility of the more aggressive cell type to treatment with statins (Fig. 2).

**Figure 3:**
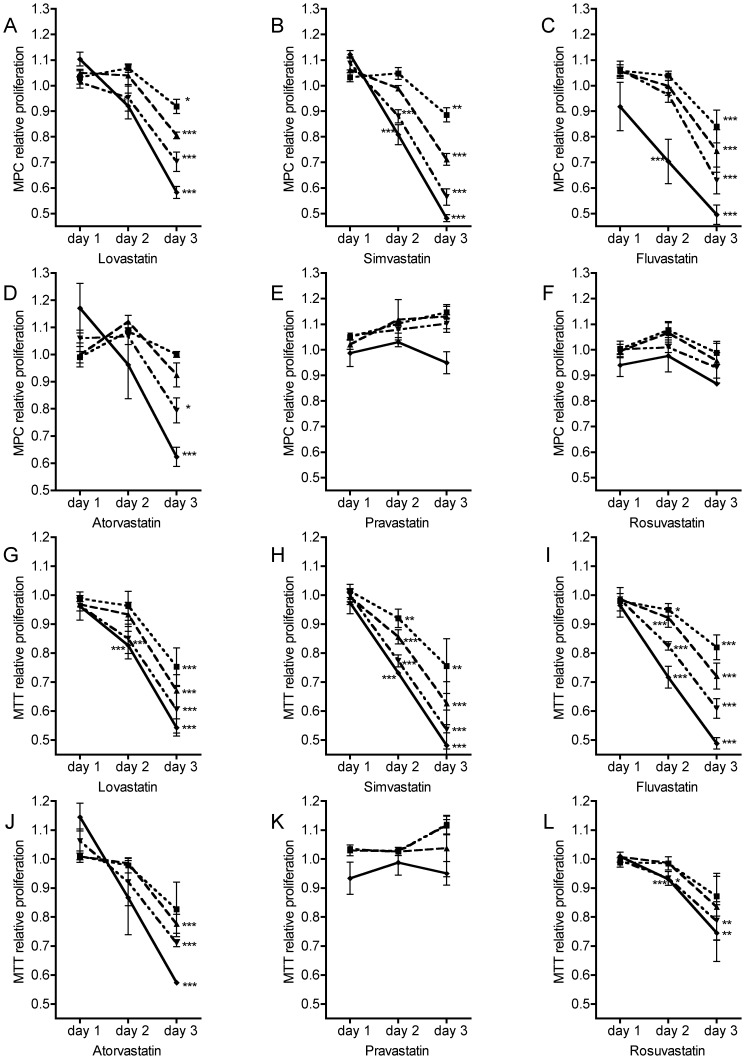
Relative proliferation of MPC and MTT under statin treatment. MPC (A–F) and MTT (G–K) were treated with ▪ 6.25 µM, ▴ 12.50 µM, ▾ 25.00 µM, and ⧫ 50.00 µM of atorvastatin, fluvastatin, lovastatin, pravastatin, rosuvastatin, and simvastatin for 24, 48, and 72 hours.

The anti-proliferative effect of the six statins differed significantly (p<0.0001). Lovastatin, simvastatin, and fluvastatin demonstrated highest proliferation inhibition (Fig. 3). Direct comparison of their anti-proliferative efficacies revealed higher potency of simvastatin and fluvastatin compared to lovastatin (p = 0.033; simvastatin vs. lovastatin SNK p = 0.043, fluvastatin vs. lovastatin SNK p = 0.031).

**Figure 4:**
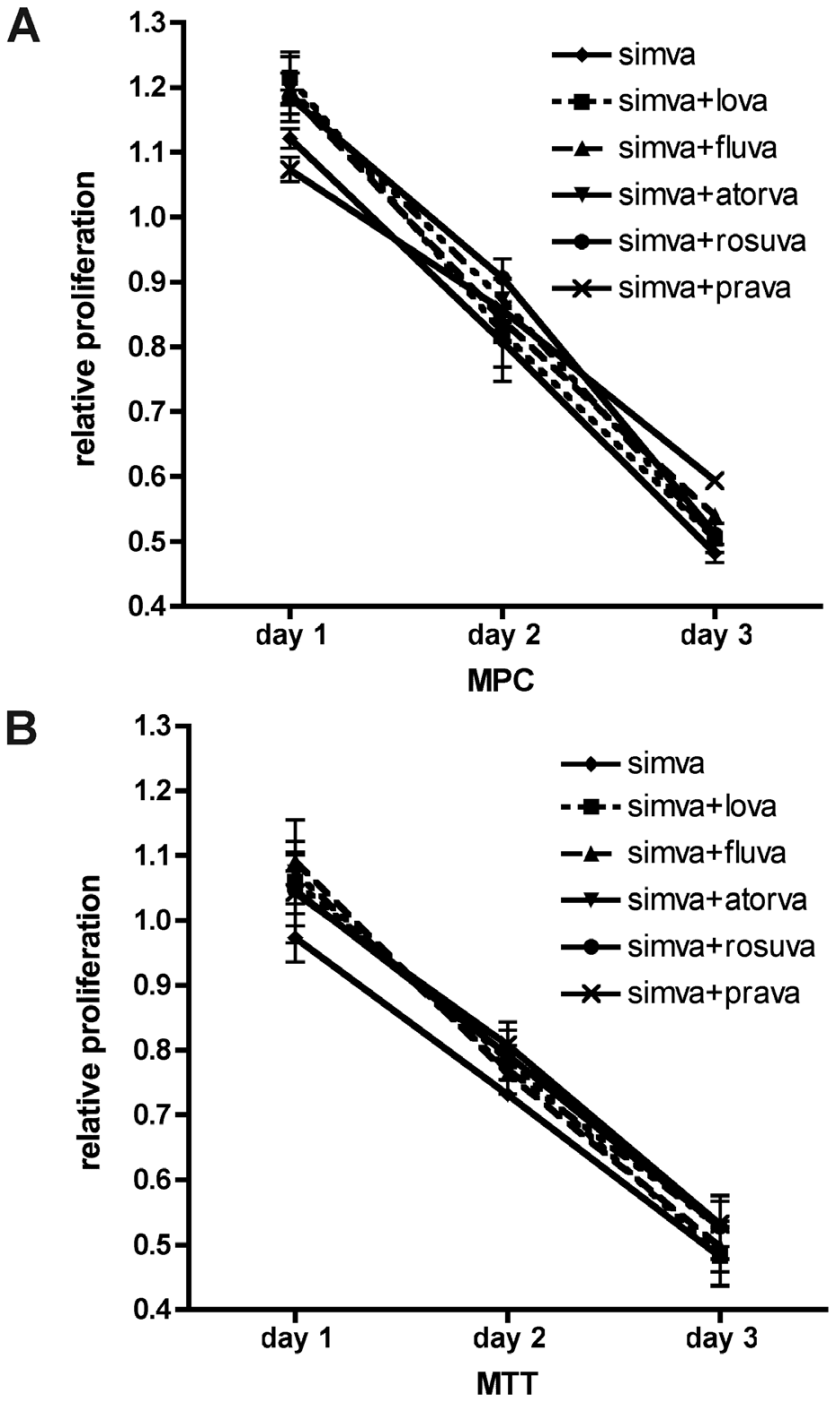
Combination of two different statins to evaluate potential additive effects on MPC or MTT. Relative viability of MPC (A) and MTT (B) at the indicated doses and durations for the most effective combined treatments including simvastatin relative to simvastatin alone.

In addition to establishing which statin is most effective in inhibiting cell proliferation in MPC and MTT, we explored whether combining two different statins might increase efficacy. Interestingly, the four most effective combinations all included simvastatin: simvastatin/lovastatin, simvastatin/fluvastatin, simvastatin/atorvastatin, and simvastatin/rosuvastatin. However, none of these combinations decreased relative viability more effectively than the three most effective statins, fluvastatin, simvastatin, and lovastatin alone (Fig. 4).

**Figure 5:**
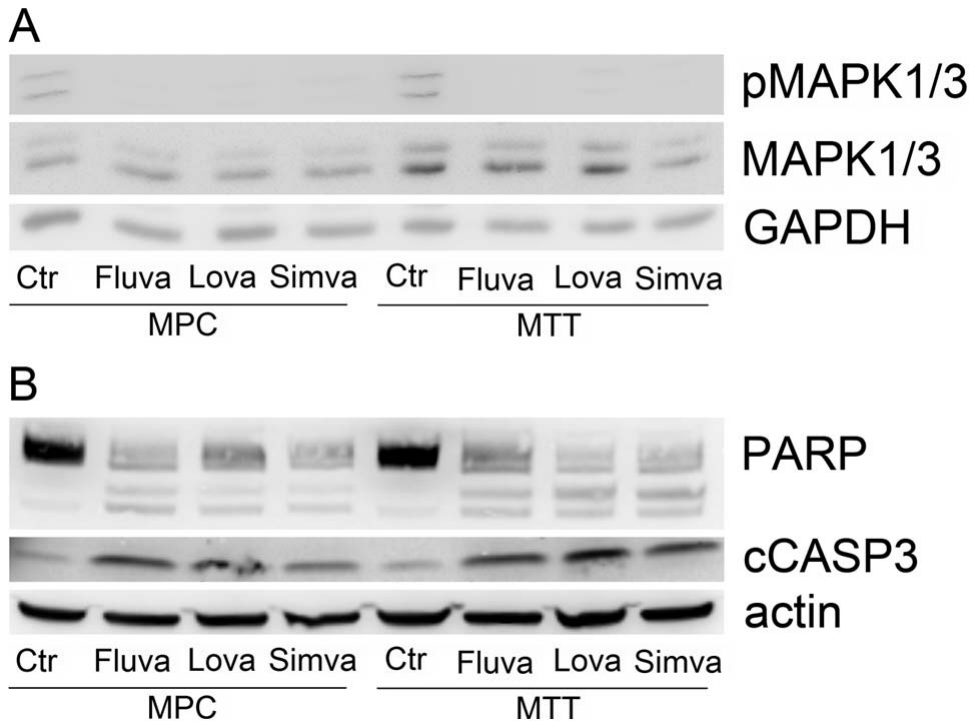
Expression of selected proteins in MPC and MTT after treatment with fluvastatin, simvastatin, or lovastatin. A. Western blot showing decreased levels of pMAPK1/3 in treated vs. untreated MPC and MTT relative to total MAPK1/3 and GAPDH. B. Western blot showing decreased levels of intact PARP (top bands) and increased levels of cleaved PARP (lower bands) in treated vs. untreated MPC and MTT. In accordance, cleaved CASP-3 was elevated in treated cells, indicating apoptosis. Cells were treated with 25 µM of the indicated statin for 48 hours.

Western blot revealed that the three most effective statins all inhibited MAPK phosphorylation at 48 h of treatment with 25 µM (Fig. 5A). In addition, fluvastatin, simvastatin, and lovastatin increased cleavage product levels of CASP-3 and PARP, indicating increased apoptosis (Fig. 5B).

**Figure 6:**
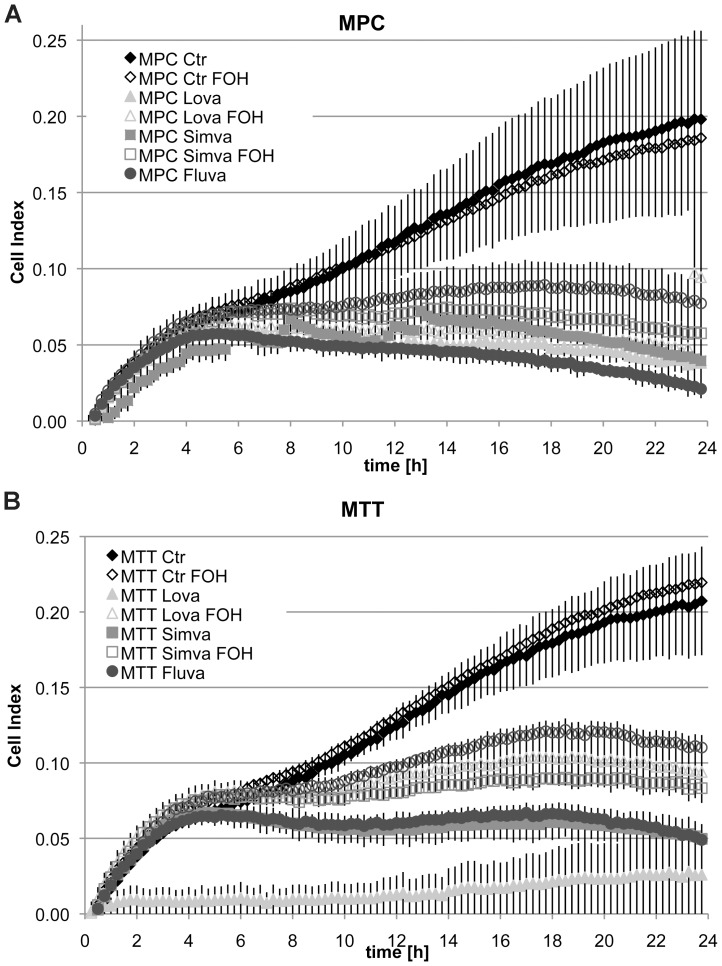
Influence of statin treatment on spontaneous cell migration. MPC (A) and MTT (B) were plated in vehicle (Ctr), 5 µM fluvastatin (Fluva), 5 µM simvastatin (Simva), 5 µM lovastatin (Lova) with or without 100 µM trans, trans farnesol (FOH) and spontaneous migration was recorded for 24.

Proliferation of neither MPC nor MTT was impacted by 6.25 µM of any tested statin after 24 or 48 h. However, spontaneous cell migration of MPC and MTT was severely inhibited by treatment with 5 µM of fluvastatin, simvastatin, and lovastatin (Fig. 6). Migratory capacity was partly rescued by addition of 100 µM trans, trans farnesol.

## Discussion

To date, no curative treatment has been established for metastatic PHEOs/PGLs. However, several new therapeutic strategies have been recently tested in model organisms [35]–[38], including lovastatin [1]. Previously, statins have been reported to decrease proliferation, survival, cell cycle progression, and migration in other cells including aggressive cancer models, amongst others by MAPK pathway inhibition [10], [25]–[27], [29], [30], [39]–[41]. However, currently evidence that statin treatment will be potent in the most aggressive type of PHEOs/PGLs, i.e. those with SDHB mutations [42], is lacking.

Our data indicate that MAPK1/3 phosphorylation is present in some SDHB-PGLs, including metastases. In agreement a previous study showed increased MAPK1/3 phosphorylation in immortalized lymphoblastoids from patients with Cowden-like syndrome and germline SDHB or SDHD gene variants or mutations [23]. In addition, several MAPK pathway genes appeared to be more highly expressed in SDHB-PGLs than in normal adrenal medulla. The exact function and potential involvement of the identified genes in SDHB-mutation mediated tumorigenesis remain to be evaluated.

In conclusion at least a subset of patients with aggressive PGLs which show MAPK1/3 phosphorylation may benefit from statin treatment. Our data indicates that this patient group may not be restricted to cluster 2 PGL patients. Determination of MAPK1/3 phosphorylation on a case by case basis in metastatic PGLs may be useful for estimation of a potential benefit of statin treatment.

In a previously published microarray based study comparing PHEOs/PGLs with different genetic backgrounds, SDHx-derived tumors showed decreased MAPK pathway gene expression relative to RET, NF1, TMEM127, and sporadic PHEOs/PGLs [21]. The discrepancy between this result and our data may be due to the fact that, relative to RET, NF1, TMEM127, and certain sporadic PHEOs/PGLs, the up-regulation of MAPK signaling genes may be marginal in SDHB PHEOs/PGLs, and thus not detectable in the absence of normal control tissue. Our microarray data revealed opposing expression patterns in SDHB and SDHD-HN PGLs with respect to MAPK signaling genes; thus grouping those samples may obscure the over-expression of MAPK-related genes in SDHB PHEOs/PGLs compared to other PHEOs/PGLs. Here we present several MAPK signaling genes that are up-regulated relative to normal adrenal medulla. Thus, we conclude that statin treatment by itself or combined with other therapeutic regimens may be of benefit in certain SDHB-derived PHEOs/PGLs.

Currently, seven different statins are on the market that – in addition to their cholesterol lowering characteristics – have been shown to interfere with several cancer relevant pathways [43], [44]. Their pharmacological actions and impact on gene expression have been shown to differ [43], [45], [46] and thus, depending on the cells to be treated, the most effective drug has to be determined. Here we show that in case of MPC and MTT, the lipophilic statins fluvastatin, simvastatin, and lovastatin considerably reduced cell proliferation, with simvastatin and fluvastatin showing slightly stronger effects. Pravastatin has been shown to be ineffective in several cancer models [47], [48], which may be due to its hydrophilic characteristics and the lack of appropriate transporters on the tumor cells. In other studies comparing the effects of several statins, simvastatin or fluvastatin appeared more effective than lovastatin [24], [30]. For further studies, consideration may be given to the fact that the pharmacokinetic characteristics of fluvastatin have been reported to be preferable to those of simvastatin and lovastatin [45].

Interestingly, the more aggressive MTT cells appeared to be more sensitive to statin treatment, which indicates that more aggressive cells may be more receptive to the anti-proliferative effects of statins. The difference in mechanism rendering MTT more susceptible remains to be elucidated.

The maximum statin concentration reported in human blood after oral administration (12.3 µM) [49] was in the range of our third highest concentration (12.5 µM), which significantly decreased cell proliferation in MPC and MTT at 48 or 72 hours of treatment with fluvastatin, lovastatin, and simvastatin. Holstein et al. reported that up to 415 mg/m^2^ orally every six hours over four days were well tolerated by patients with severe malignancies [49]. However, blood statin levels did not increase in a linear manner, and thus oral administration may not be the best option to achieve high tissue concentrations. Optimal administration techniques to achieve the targeted concentrations will have to be determined. Negligible effects of statins on normal cells compared to cancerous cells have been reported [24], [50].

At non-toxic concentrations, achievable by oral administration, statins have been reported to exhibit another anti-cancer effect, the inhibition of cell migration and invasion [9], [47], [51]. For this reason we tested the consequence of the effective statins, lovastatin, simvastatin, and fluvastatin on spontaneous cell motility of MPC and MTT at 5 µM. Our proliferation assays showed no reduction in proliferation at 6.5 µM within 24 hours of treatment. Lovastatin, fluvastatin, and simvastatin effectively inhibited migration of MPC and MTT within 24 hours. The migration-inhibition appeared to be at least partly dependent on farnesyl depletion, since presence of trans, trans farnesol partly rescued migratory capacity. However, even a relatively high concentration of 100 µM trans, trans farnesol did not entirely reverse the anti-migratory effect of the three statins. Thus, farnesylation-independent effects of statins on MPC and MTT cannot be excluded. In a previous study, geranylgeranyl pyrophosphate has been shown to be more effective in reversing the effect of statins than farnesylpyrophosphate [24]. In addition, non-mevalonate pathway dependent statin effects on cancer cells have also been described [43].

Since oral administration of statins does not yield concentrations as high as used here to achieve the best anti-proliferative effects in vitro, combined administration of other drugs appears to be a promising strategy. The multikinase inhibitors sunitinib and sorafenib also affect the MAPK pathway and have been shown to be effective in progressive metastatic PGLs [52], [53]. Sunitinib treatment showed beneficial clinical effects in 8 out of 17 patients with progressive metastatic PGLs. Interestingly, 6 of the responders carried SDHB-mutations. However, as most anti-neoplastic drugs, the side effects of multikinase inhibitors can be severe and nine of the 17 patients treated with sunitinib had to discontinue treatment or decrease the dose eventually.

A synergistic effect of fluvastatin and sorafenib has been presented for melanoma cells in vitro [54]. This holds the potential of decreasing multikinase inhibitor concentrations, which may relieve the severity of side effects while anti-tumor performance is maintained. However, further studies are mandated to establish whether combination of multikinase inhibitors with statins may have additive effects in aggressive PGLs. As previously reported, combined inhibition of PI3K/AKT and mTORC1/2 signaling has been suggested as promising therapeutic regimen for certain PGLs [1].

In conclusion, statins may be of benefit alone or in combination with other therapeutic regimens in patients with aggressive PHEOs/PGLs which show MAPK pathway activity.

## Supporting Information

Table S1 **Sample numbers from** **Figure 1A** **with corresponding Chip Identifiers and sample identifiers from Shankavaram & Fliedner et al. 2013 Neoplasia 15: 435–447.** Patient information and link to deposited microarray data are given in the original article.
(XLSX)
